# The sustained psychological impact of coronavirus disease 2019 pandemic on hospital workers 2 years after the outbreak: a repeated cross-sectional study in Kobe

**DOI:** 10.1186/s12888-023-04788-8

**Published:** 2023-05-04

**Authors:** Haruko Fukushima, Hissei Imai, Chisato Miyakoshi, Atsumi Naito, Kyohei Otani, Kunitaka Matsuishi

**Affiliations:** 1 Department of Psychiatry, Kobe City Medical Center General Hospital, Kobe, Japan; 2 Health Promotion and Behavior, Graduate School of Medicine, School of Public Health, Kyoto University, Ohashi Clinic, Kyoto, Japan; 3 Department of Research Support, Center for Clinical Research and Innovation, Kobe City Medical Center General Hospital, Kobe, Japan; 4 Department of Psychiatry, Kakogawa Central City Hospital, Kakogawa, Japan

**Keywords:** Burnout, COVID-19 pandemic, Healthcare workers, Psychological distress, Questionnaire

## Abstract

**Background:**

Healthcare workers who are exposed to coronavirus disease 2019 are psychologically distressed. This study aimed to evaluate the mental health outcomes of hospital workers 2 years after the outbreak of coronavirus disease 2019 and to identify changes in the stress of hospital workers and predicted risk factors.

**Methods:**

This survey was conducted 2 years after the initial evaluation performed under the first emergency declaration of the coronavirus disease 2019 pandemic among hospital workers at the same hospital in an ordinance-designated city in Japan from June to July 2022. Sociodemographic data, 19 stress-related question responses, the Impact of Event Scale-Revised, and the Maslach burnout inventory-general survey were collected. Multiple regression models were used to identify factors associated with each of the mental health outcomes 2 years after the coronavirus disease 2019 outbreak.

**Results:**

We received 719 valid responses. Between 2020 and 2022, hospital workers’ anxiety about infection decreased, whereas their exhaustion and workload increased. Multiple regression analysis revealed that 2 years after the coronavirus disease 2019 outbreak, nurses and young people were at a higher risk of experiencing stress and burnout due to emotional exhaustion, respectively.

**Conclusions:**

This is the first study to examine the long-term stress of hospital workers measured in Japan. Exhaustion and workload were worsened 2 years into the pandemic. Therefore, health and medical institutions should continuously monitor the physical and psychological health of staff members.

## Background

More than 100 million infections and 2 million deaths have been recorded worldwide due to the devastating global health crisis brought on by the novel coronavirus disease 2019 (COVID-19) pandemic in 2020 [1]. As of November 2022, there were seven waves in Japan, with 22.7 million infections and more than 47,000 deaths [2]. The impact of the COVID-19 pandemic has been devastating for patients and also healthcare workers. Working in a large tertiary hospital during the COVID-19 pandemic has been found to be stressful or traumatic for many healthcare workers [3]. On March 3, 2020, Kobe City Medical Center General Hospital (KCGH) admitted its first COVID-19-infected patient in Kobe. We have previously reported the psychological impact of the COVID-19 pandemic on hospital workers under the first emergency declaration of the COVID-19 pandemic in Japan [4]. Although early studies have reported elevated rates of chronic stress, anxiety, and job burnout among healthcare workers, the longer-term impact of the pandemic is still largely unknown [5, 6].

The few studies, which explored the short-term impact of the pandemic on healthcare workers provide various results. A study on Argentinean healthcare workers showed a deterioration of self-perceived job performance and increased prevalence of depression and anxiety over a 4-month period [7]. In contrast, a study among intensive care unit nurses in Belgium showed that they had improved depression, anxiety, and somatization over a 2-month period [8].

To date, limited data is available on the psychological impact of the COVID-19 pandemic on healthcare workers over a more prolonged period. A repeated cross-sectional study on intensive care physicians in a COVID-19 hub hospital in Central Italy reported sustained high levels of occupational stress, anxiety, and depression, low satisfaction, and burnout over 1 year since the pandemic onset [9]. Some longitudinal studies have been reported during previous pandemics. For example, during the outbreak of severe acute respiratory syndrome (SARS) in 2003, healthcare professionals experienced high levels of posttraumatic stress symptoms that lasted for approximately 3 years [10, 11]. Future studies should analyze the long-term effects of the COVID-19 pandemic on the mental health of hospital workers [12]. Patient-related stressful situations in these workers appear to be associated with burnout and posttraumatic stress disorder [13]. Therefore, understanding the enduring psychological effects of working during the COVID-19 pandemic is important since it involves the well-being of many hospital workers and, in turn, the effectiveness and safety of the care provided to patients [14].

On March 3, 2020, KCGH admitted its first COVID-19 infected patient in Kobe, and by the end of July 2022, 1,610 patients with severe COVID-19 had been admitted. During this period, seven waves of the pandemics were recorded, and we believe that hospital workers experienced more severe physical and psychological stress than ever before. Therefore, this study aimed to assess the psychological impact of the COVID-19 pandemic on hospital workers 2 years after the outbreak and to identify personal and job-related factors that might have increased the risk of developing adverse mental health outcomes.

## Methods

### Study setting and participants

This was a two-point cross-sectional study targeting different populations while they were employed at the same hospital. Specifically, it represents the second evaluation conducted on the hospital workers of the KCGH. They were first assessed during the first emergency declaration of the COVID-19 pandemic (from April 16 to June 8, 2020). All staff working at KCGH during the COVID-19 pandemic were asked to participate. Employees who accepted to participate and signed a written consent form comprised the study’s sample. The findings of the first assessment are presented in detail elsewhere [4]. Four categories (Factor 1: anxiety about infection, Factor 2: exhaustion, Factor 3: workload, Factor 4: feeling of being protected) were identified as significant factors in the previous study [4]. Two years after the first assessment (from June 17 to July 31, 2022), hospital workers of the KCGH were invited to re-assess their psychological status. Similarly, to the first assessment, the evaluation conducted in 2022 was performed using self-rated scales hosted on a web-based survey (Google Forms), where participants could complete the online questionnaires using their personal computers, smartphones, or other mobile devices. This study was conducted under the same circumstances as the previous study, both respondents were allowed to be anonymous, therefore the data were unlinked. The study description and invitation to participate, as well as the link to the online questionnaires, were put up all over the hospital and sent via e-mail to all hospital workers. All employees were notified of the study information and purpose in accordance with the International Ethical Guidelines for Health-related Research Involving Humans; the disclosure document was sent via e-mail, and the employees were provided the opportunity to refuse participation. The study protocol was approved by the Ethics Committee of KCGH (no. zn220903).

### Content of the questionnaire

The questionnaire explained the study’s purpose, which was to examine the stress experienced by hospital workers during the COVID-19 pandemic. It comprised items covering sociodemographic characteristics, stress-related questions associated with the COVID-19 outbreak, the Impact of Event Scale-Revised (IES-R), and the Maslach burnout inventory-general survey (MBI-GS).

Personal characteristics included gender, age group, job, and work environment during the COVID-19 pandemic. The respondents were asked if they had experienced the Great Hanshin-Awaji Earthquake and whether they had participated in the Disaster Medical Assistance Team (DMAT). The work environment was categorized into frontliner (a respondent working in the ward for COVID-19 infection and the fever consultation center) and non-frontliner (a respondent working in any other place).

Overall, 19 questions related to stress were included (Table 1). The respondents indicated how frequently they experienced the conditions covered by these items during the pandemic using a 4-point Likert scale. The 19 items used in our study were based on similar items in a stress questionnaire used to study an influenza pandemic (H1N1) 2009 [15, 16].

**Table 1:** Stress-related questions associated with the COVID-19 outbreak

Questions		0 *Never*	1 *Rarely*	2 *Sometimes*	3 *Always*
Q1	I felt anxious about being infected.				
Q2	I felt anxious about infecting my family.				
Q3	I felt burdened by the increased quantity of work.				
Q4	I felt burdened by the changed quality of work.				
Q5	I felt anxious about being infected during commuting.				
Q6	I felt lacking knowledge about prevention and protection.				
Q7	I felt lacking in knowledge about infectiosity and virulence.				
Q8	I felt avoided by others.				
Q9	I felt protected by my country or local government.				
Q10	I felt protected by my hospital.				
Q11	I felt anxious about compensation in the case of being infected.				
Q12	I felt hesitant to work.				
Q13	I felt isolated.				
Q14	I felt an elevated mood.				
Q15	I had insomnia.				
Q16	I was exhausted physically.				
Q17	I was exhausted mentally.				
Q18	I felt motivated to work.				
Q19	I felt I had no choice but to work due to obligation.				

COVID-19, coronavirus disease 2019

The IES-R is a self-report measure of current subjective distress in response to a specific traumatic event. This 22-item scale comprises three subscales, which are representative of the major symptom clusters of post-traumatic stress as follows: intrusion, avoidance, and hyperarousal [17]. The respondent was asked to report the degree of distress experienced for an item in the past 7 days. The 5 points on the scale are as follows: 0 (not at all), 1 (a little bit), 2 (moderately), 3 (quite a bit), and 4 (extremely). The reliability and validity of the Japanese version of the IES-R have been verified. A cut-off score of 24/25 defined posttraumatic stress disorder (PTSD) as a clinical concern [18].

Burnout was assessed using the MBI-GS [19], which is a modified version of the original MBI [20] found to be reliable and valid across multiple cultural settings and occupations, including healthcare professionals [21–24]. It comprises 16 items with 7 response options that are answered on a Likert scale, from 0 (never) to 6 (every day). The Japanese version of the MBI-GS has been validated for the Japanese population [25–28]. This questionnaire contains three subscales that evaluate the three major domains of burnout, namely, emotional exhaustion, cynicism, and professional efficacy. The cutoff values for burnout are as follows: An Exhaustion score of > 3.5 and a Cynicism score of > 3.5 or an Exhaustion score of > 3.5 and a Professional Efficacy score of < 2.5 [29–31].

### Statistical analysis

The participants’ characteristics were summarized as numbers and percentages and mean and standard deviation (*SD*) for the categorical and continuous variables, respectively.

During the H1N1 influenza pandemic, we performed an exploratory factor analysis and identified four factors for evaluation (anxiety about infection, exhaustion, workload, and feeling of being protected) using a stress-related questionnaire survey among hospital workers [15, 16]. Confirmatory factor analysis (CFA) was conducted, and we confirmed the same four-factor structure tested by the stress-related survey during the first wave of the COVID-19 pandemic [4]. Factor 1 comprised items 1, 2, 5, 6, 7, and 11, representing “anxiety about infection.” Factor 2 included items 14, 15, 16, and 17, indicating “exhaustion.“ Factor 3 consisted of items 3 and 4, which represented “workload.” Finally, Factor 4, which indicated a “feeling of being protected,” was based on items 9 and 10.

The total score of questionnaire items for each of the four factors was calculated. Here, each factor’s score, the IES-R, and MBI-GS were compared between strata of each personal characteristic using a Student’s *t*-test or analysis of variance. We also evaluated the association of personal characteristics with each score, the IES-R, and MBI-GS using multiple linear regression models. However, participants with missing data were excluded from the regression analyses. We compared the baseline characteristics of participants with and without burnout using the χ^2^ difference test. Results for each of the four factors of the 19 stress-related questions and the IES-R in 2020 and 2022 were compared using Welch’s t-test.

All analyses were performed using the R statistical software (version 4.1.0, Vienna, Austria) and SPSS 27 statistical package (IBM Corp. Released 2021, IBM SPSS Statistics for Windows, Version 27.0., Armonk, NY).

## Results

Overall, 798 employees completed the questionnaire from June 17, 2022 to July 31, 2022. Of these responses, 79 contained at least one missing answer, leaving 719 questionnaires (response rate: 22.5%) for analysis. In contrast, 1,111 healthcare workers participated in the first assessment from June 17, 2020 to July 31, 2022. Of the responses provided, 160 contained at least one missing answer, leaving 951 questionnaires (response rate: 29.6%) for analysis. Table 2 presents the characteristics of the valid respondents in 2020 and 2022. Jobs classified as medical doctor, nurse, or others (radiologic technologists, clinical laboratory technicians, pharmacists, dieticians, social workers, physical therapists, occupational therapists and speech therapists, biomedical equipment technicians, office workers, clinical clerks, guards, and janitors).

**Table 2:** The characteristics of the valid respondents

		First Assessment		Second Assessment	
		*n*	(%)	*n*	(%)
Total valid respondents		951		719	
Gender					
Male		311	(32.7)	211	(29.3)
Female		640	(67.3)	508	(70.7)
Age group (years)					
20–29		311	(32.7)	193	(26.8)
30–39		244	(25.7)	179	(24.9)
40–49		223	(23.4)	164	(22.8)
50–59		129	(13.6)	143	(19.9)
60+		44	(4.6)	40	(5.6)
Job					
Medical doctor		157	(16.5)	105	(14.6)
Nurse		343	(36.1)	166	(23.1)
Others		451	(47.4)	448	(62.3)

Job classified as medical doctor, nurse, or others (radiologic technologists, clinical laboratory technicians, pharmacists, dieticians, social workers, physical therapists, occupational therapists and speech therapists, biomedical equipment technicians, office workers, clinical clerks, guards, and janitors)

### Mental health outcomes 2 years after the pandemic onset

Each of the four factors in the 19 stress-related questions and the IES-R at first (2020) and second (2022) assessment points were compared using Welch’s t-test (Table 3). Factor 1, “anxiety about infection,” was significantly lower in the second assessment point than the first (degree of freedom (df) = 1536.51, p < .001). Factor 2, “exhaustion,” and factor 3, “workload,” were significantly higher in the second than in the first (exhaustion: df = 1590.31, p < .001; workload: df = 1532.43, p < .001). Factor 4, which is the “feeling of being protected,” was significantly higher in the second than in the first (df = 1578.14, p < .001). The score in the second was higher than that in the first regarding the IES-R, although the difference was not significant.

**Table 3:** Comparison of stress-related questions and IES-R in the first (2020) and second (2022) assessment points

	First Assessment		Second Assessment		Welch’s two-sample *t*-test			
	Mean	SD	Mean	SD	df		*t*	*p*
F1 (anxiety about infection)								
	10.07	3.03	9.08	3.06	1536.51		6.58	< 0.001
F2 (exhaustion)								
	4.20	2.51	5.11	2.36	1590.31	-7.59		< 0.001
F3 (workload)								
	2.38	1.52	3.25	1.55	1532.43	-11.60		< 0.001
F4 (feeling of being protected)								
	2.05	1.21	2.44	1.16	1578.14	-6.64		< 0.001
IES-R								
	12.71	13.35	13.93	15.45	1416.13	-1.69		0.91

Abbreviations: df, degree of freedom; IES-R, Impact of Event Scale-Revised; SD, standard deviation

### Regression analysis

The data of 719 (22.5%) participants were included in the regression analysis. Table 4 presents the estimated associations of the sociodemographic characteristics with the total score for each of the four factors and the IES-R. The independent variables were gender, age group, job, exposure to COVID-19, the experience of the Great Hanshin-Awaji Earthquake, and experience of engaging in DMAT. We investigated whether the experience of the Great Hanshin-Awaji Earthquake would be a factor of vulnerability in re-experiencing the trauma and if the experience of DMAT participation would be a protective factor through prior education. The dependent variables were factors 1, 2, 3, and 4 and the IES-R score.

**Table 4:** Regression analysis

								F1(anxiety about infection): Q1,Q2,Q5,Q7,Q6,Q11													F2(exhaustion): Q16,Q17,Q15,Q14											
												Univariable				Multivariable											Univariable		Multivariable			
			*N*		(%)			mean		(*SD*)		p-value				β		p-value			mean			(*SD*)			p-value		β		p-value	
Sex																																
Male			211		(29.3)			8.03		(3.16)		< .001				-					4.82			(2.40)			.036		-			
Female			508		(70.7)			9.52		(2.92)						1.175		< .001			5.22			(2.34)					0.371		.097	
Age (years)																																
20–29			193		(26.8)			9.32		(3.29)		.59				-					5.25			(2.51)			.54		-			
30–39			179		(25.0)			8.85		(3.20)						-0.209		.507			5.08			(2.44)					-0.042		.866	
40–49			164		(22.8)			9.00		(2.72)						-0.114		.726			5.19			(2.19)					0.089		.733	
50–59			143		(19.9)			9.22		(3.07)						0.231		.516			4.97			(2.29)					-0.031		.914	
60+			40		(5.6)			8.82		(2.60)						0.101		.850			4.62			(2.25)					-0.307		.470	
Job																																
Medical doctor			105		(14.6)			7.35		(2.90)		< .001				-					4.82			(2.32)			.18		-			
Nurse			166		(23.1)			9.39		(2.58)						1.300		.002			5.36			(2.22)					0.300		.370	
Others			448		(62.3)			9.37		(3.14)						1.689		< .001			5.08			(2.42)					0.199		.472	
Exposure to COVID-19																																
Non-frontliner			528		(73.4)			9.01		(3.01)		.314				-					5.02			(2.34)			.094		-			
Frontliner			191		(26.6)			9.27		(3.21)						0.68		.010			5.35			(2.41)					0.366		.082	
Experience of the Great Hanshin-Awaji Earthquake																																
N			575		(80.0)			9.14		(3.12)		.337				-					5.18			(2.39)			.081		-			
Y			144		(20.0)			8.86		(2.81)						-0.536		.076			4.80			(2.26)					-0.371		.124	
Experience of engagement in DMAT																																
N			696		(96.8)			9.09		(3.06)		.541				-					5.12			(2.37)			.450		-			
Y			23		(3.20)			8.70		(3.36)						0.132		.836			4.74			(2.32)					-0.143		.780	
		**F3(workload): Q4,Q3**												**F4(feeling of being protected): Q10,Q9**											**IES-R**							
						**Univariable**			**Multivariable**								**Univariable**		**Multivariable**									**Univariable**		**Multivariable**		
		**mean**		(***SD***)		**p-value**			**β**		**p-value**			**mean**	(***SD***)		**p-value**		**β**			**p-value**			**mean**	(***SD***)		**p-value**		**β**		**p-value**
Sex																																
Male		3.24		(1.57)		.845			-					2.55	(1.14)		.108		-						14.37	(15.45)		.623		-		
Female		3.26		(1.54)					-0.07		.618			2.39	(1.17)				-0.13			.239			13.75	(15.47)				-1.68		.251
Age (years)																																
20–29		3.26		(1.62)		.24			-					2.32	(1.24)		.20		-						16.23	(17.61)		.14		-		
30–39		3.36		(1.65)					0.16		.301			2.39	(1.07)				0.04			.763			12.86	(14.84)				-4.15		.012
40–49		3.24		(1.45)					0.17		.292			2.47	(1.14)				0.12			.332			14.04	(15.16)				-2.86		.091
50–59		3.28		(1.43)					0.36		.044			2.55	(1.18)				0.18			.194			12.27	(13.77)				-4.79		.010
60+		2.73		(1.40)					-0.15		.582			2.70	(1.20)				0.30			.152			13.08	(13.02)				-3.69		.184
Job																																
Medical doctor		3.07		(1.57)		< .001			-					2.62	(1.18)		.22		-						11.70	(15.15)		.27		-		
Nurse		3.78		(1.45)					0.83		< .001			2.41	(1.02)				-0.10			.544			14.01	(14.41)				2.61		.232
Others		3.10		(1.54)					0.25		.152			2.40	(1.21)				-0.19			.169			14.42	(15.88)				3.70		.041
Exposure to COVID-19																																
Non-frontliner		3.03		(1.50)		< .001			-					2.45	(1.15)		.495		-						13.47	(15.45)		.189		-		
Frontliner		3.87		(1.51)					0.75		< .001			2.39	(1.21)				-0.08			.413			15.19	(15.42)				1.87		.173
Experience of the Great Hanshin-Awaji Earthquake																																
N		3.31		(1.55)		.056			-					2.40	(1.18)		.107		-						13.85	(15.87)		.781		-		
Y		3.03		(1.51)					-0.34		.026			2.58	(1.10)				0.12			.324			14.25	(13.70)				1.67		.289
Experience of engagement in DMAT																																
N		3.25		(1.54)		.875			-					2.43	(1.16)		.591		-						13.88	(15.45)		.655		-		
Y		3.30		(1.74)					0.08		.803			2.57	(1.20)				0.03			.919			15.35	(15.80)				1.34		.687

Adjusted for sex, age group, job, exposure to COVID-19, the experience of the Great Hanshin-Awaji Earthquake, and experience of engagement in DMAT

Abbreviations: COVID-19, coronavirus disease 2019; DMAT, disaster medical assistance team; IES-R, Impact of Event Scale-Revised; SD, standard deviation

Regarding gender, females reported higher levels of anxiety than males for factor 1, “anxiety about infection” (β = 1.18, p < .001). Nurses and others had higher levels of anxiety about infection than medical doctors for the job category (nurses: β = 1.30, p = .002; others: β = 1.69, p < .001). Related to exposure to COVID-19, the frontliners felt that they had higher levels of anxiety than the non-frontliners (β = 0.68, p = .010).

For factor 2, “exhaustion,” the scores did not vary by gender, age, job, exposure to COVID-19, experiences with the Great Hanshin-Awaji Earthquake, and experience of engaging in DMAT.

For factor 3, “workload,” workers in their 50s reported more demanding workloads than those in their 20s (β = 0.36, p = .044). Nurses reported that they had a greater workload than medical doctors (β = 0.83, p < .001). Concerning exposure to COVID-19, frontliners felt that they had a higher workload than non-frontliners (β = 0.75, p < .001). Workers who experienced the Great Hanshin-Awaji Earthquake felt less workload than workers who did not experience it (β = − 0.34, p = .026).

For factor 4, “feeling of being protected,” the scores did not vary by gender, age, job, exposure to COVID-19, experiences with the Great Hanshin-Awaji Earthquake, and experience of engaging in DMAT.

The mean (*SD*) total score on the IES-R in the 719 participants was 13.9 (15.5), ranging from 0 to 88. Notably, 150 (20.9%) respondents were screened positive on clinical concerns for PTSD. The total IES-R scores varied by age and job in the regression analysis. The total IES-R scores of workers in their 30 and 50 s were lower than those of workers in their 20s (30s: β = -4.15, p = .012; 50s: β = -4.79, p = .010). Examined by job category, the total IES-R score of others was higher than that of medical doctors (β = 3.70, p = .041).

### Burnout

Overall, 123 (17.1%) employees satisfied the Japanese Version of the MBI-GS burnout criteria. Table 5 summarizes the results of the detailed demographic data for the burnout group. Thirty-three (15.6%) males and 90 (17.7%) females experienced burnout, but no significant difference was found in the prevalence of burnout between males and females (p = .501). Of the 166 nurses and 448 others, 38 (22.9%) and 67 (15.0%) experienced burnout, respectively. Significant differences were not observed between the two groups in the proportion of age (p = .126), job (p = .068), and exposure to COVID-19 (p = .456).

**Table 5:** Comparison of characteristics between the burnout and nonburnout groups

		Burnout	Nonburnout	Total	p-value^*^
		n = 123 (17.1%)	n = 596 (82.9%)		
Gender	Male	33 (15.6)	178 (84.4)	211	0.501
	Female	90 (17.7)	418 (82.3)	508	
Age (years)	20–29	39 (20.2)	154 (79.8)	193	0.126
	30–39	36 (20.1)	143 (79.9)	179	
	40–49	21 (12.8)	143 (87.2)	164	
	50–59	24 (16.8)	119 (83.2)	143	
	60+	3 (7.5)	37 (92.5)	40	
Job	Medical doctor	18 (17.1)	87 (82.9)	105	0.068
	Nurse	38 (22.9)	128 (77.1)	166	
	Others	67 (15.0)	381 (85.0)	448	
Exposure to COVID-19	Non-frontliner	87 (16.5)	441 (83.5)	528	0.456
	Frontliner	36 (18.8)	155 (81.2)	191	

^*^ chi-square test;

Abbreviation: COVID-19, coronavirus disease 2019

Table 6 lists the estimated associations of the sociodemographic characteristics with the MBI-GS subscale scores. The independent variables were sex, age group, job, exposure to COVID-19, experience of the Great Hanshin-Awaji Earthquake, and experience of engagement in DMAT. The dependent variables were emotional exhaustion, cynicism, and professional efficacy.

**Table 6:** MBI-GS subscale scores

		MBI-GS (emotional exhaustion): Q1,Q2,Q3,Q4,Q6						MBI-GS (cynicism):Q5,Q7,Q10,Q11,Q12,Q16						MBI-GS(professional efficacy): Q8,Q9,Q13,Q14,Q15				
				Univariable	Multivariable					Univariable	Multivariable					Univariable	Multivariable	
		mean	(*SD*)	p-value	β	p-value		mean	(*SD*)	p-value	β	p-value		mean	(*SD*)	p-value	β	p-value
Sex																		
Male		2.51	(1.61)	0.001	-			2.42	(1.22)	< 0.001	-			1.68	(1.30)	0.238	-	
Female		2.98	(1.68)		1.72	0.025		1.99	(1.10)		-1.05	0.096		1.81	(1.43)		0.20	0.757
Age (years)																		
20–29		3.31	(1.66)	< 0.001	-			2.03	(1.09)	0.013	-			2.13	(1.54)	< 0.001	-	
30–39		2.88	(1.68)		-1.57	0.069		2.11	(1.16)		0.15	0.835		1.74	(1.32)		-1.92	0.008
40–49		2.76	(1.66)		-2.22	0.013		2.05	(1.17)		-0.01	0.987		1.74	(1.43)		-1.91	0.011
50–59		2.46	(1.53)		-3.80	< 0.001		2.15	(1.08)		0.55	0.493		1.42	(1.15)		-3.66	< 0.001
60+		2.03	(1.64)		-5.83	< 0.001		2.71	(1.42)		3.55	0.003		1.54	(1.21)		-3.00	0.015
Job																		
Medical doctor		2.70	(1.52)	< 0.001	-			2.90	(1.24)	< 0.001	-			1.78	(1.32)	< 0.001	-	
Nurse		3.41	(1.56)		1.71	0.136		1.93	(1.00)		-4.91	< 0.001		2.15	(1.46)		1.08	0.263
Others		2.66	(1.70)		− 0.96	0.313		2.00	(1.11)		-4.85	< 0.001		1.63	(1.36)		-0.82	0.303
Exposure to COVID-19																		
Non-frontliner		2.78	(1.68)	0.120	-			2.10	(1.17)	0.681	-			1.70	(1.38)	0.036	-	
Frontliner		3.00	(1.63)		0.45	0.534		2.14	(1.10)		− 0.15	0.798		1.95	(1.40)		0.72	0.233
Experience of the Great Hanshin-Awaji Earthquake																		
N		2.87	(1.67)	0.274	-			2.12	(1.17)	0.843	-			1.76	(1.39)	0.778	-	
Y		2.70	(1.69)		0.54	0.0510		2.10	(1.10)		0.00	0.998		1.80	(1.38)		1.45	0.037
Experience of engagement in DMAT																		
N		2.84	(1.67)	0.589	-			2.11	(1.14)	0.681	-			1.77	(1.40)	0.891	-	
Y		2.65	(1.68)		0.424	0.808		2.24	(1.46)		-0.24	0.864		1.73	(1.21)		0.13	0.929

Abbreviations: COVID-19, coronavirus disease 2019; DMAT, disaster medical assistance team; MBI-GS, Maslach burnout inventory-general survey; SD, standard deviation

In the regression analysis, significant differences were found between males and females in emotional exhaustion, with females presenting higher scores than males (β = 1.72, p = .025). Additionally, significant interaction effects were found between emotional exhaustion and age, where emotional exhaustion was greater in workers in their 20s than in those aged ≥ 40 (40s: β = -2.22, p = .013; 50s: β = -3.80, p < .001; 60s: β = -5.83, p < .001).

Significant differences were found between cynicism and age, with workers in their 60s reporting feeling more cynicism than those in their 20s (β = 3.55, p = .003). Additionally, significant interaction effects were observed between cynicism and job. Medical doctors had higher scores than nurses and others (nurses: β = -4.91, p < .001; others: β = -4.85, p < .001).

Significant differences were observed between professional efficacy and age, with professional efficacy scores being higher in workers in their 20s than in those in their 30s, 40s, 50s, and 60s (30s: β = -1.92, p = .008; 40s: β = -1.91, p = .011; 50s: β = -3.66, p < .001; 60s: β = -3.00, p = .015). Regarding the experience of the Great Hanshin-Awaji Earthquake, workers who experienced it felt more professional efficacy than those who did not (β = 1.45, p = .037).

## Discussion

This study assessed the psychological status of hospital workers 2 years after the onset of the COVID-19 pandemic. The KCGH has been designated for the severe COVID-19 hospital since the beginning of the pandemic, and hospital care workers have continually provided care to both patients with COVID-19 and all other patients presenting with various medical and surgical conditions in a very difficult context characterized by the seven subsequent pandemic waves. We found that the mental health of hospital care workers deteriorated over the 2 years since the COVID-19 pandemic onset. Overall, we found that hospital workers are emotionally exhausted because of the great workload during the past 2 years, despite their perception of decreased infection anxiety and a sense of increased protection compared with previously. The study indicated that among healthcare workers, women, nurses, and frontline workers still faced multiple high-risk factors. We also found that healthcare workers in their 20s were emotionally exhausted, which is a risk factor for burnout.

### Stress and gender

Here, females experienced higher levels of anxiety than males. In contrast, “exhaustion,” “workload,” “feeling of being protected,” and the IES-R scores did not significantly differ between genders. Similar results have also been previously reported. The female gender has been consistently associated with higher levels of anxiety [32–38], whereas no consistent association has been found with PTSD [39]. Therefore, women were more susceptible to anxiety and appeared to require more attentive care.

### Stress and age

Here, the IES-R scores were significantly higher for workers in their 20s than for those in their 30 and 50 s. Other studies also showed that younger healthcare workers had a greater risk of developing PTSD [3, 11, 32, 40, 41]. Young workers might be more vulnerable when they face difficult situations such as patients suffering and dying from COVID-19, especially in cases where they are unable to provide the standard medical care due to limited resources [42]. Therefore, managers and executives should consider that younger workers are prone to stress. Since younger workers require care, it is important to create a workplace where they feel comfortable sharing their concerns.

### Stress and profession

Examined by job category, nurses and others were significantly more anxious about infection. The reported “workload” was significantly higher for nurses than for medical doctors. However, nurses are particularly vulnerable to psychological distress in the workplace since most of them have experienced sudden and dramatic challenges in increased workload, reassignment to other roles or duties, and infection threats during the COVID-19 pandemic. Similar results were reported in studies of the 2003 SARS outbreak [43, 44] and the pandemic (H1N1) 2009 in Japan [16]. Another study of the COVID-19 pandemic yielded similar results [4, 32, 45]. Nurses are more likely to develop anxiety [46] and stress [47]. Furthermore, the amount of time spent with infectious patients may explain the difference in job effects. Therefore, establishing a limit on the amount of time spent with infectious patients may be necessary. Moreover, if more technology could be used, such as the introduction of nursing robots, it may be crucial to allow indirect contact with infectious patients.

### Stress and place of posting

Hospital workers in high-risk environments (frontliners) experienced significantly higher levels of “anxiety about infection” and “workload” than those in low-risk work environments (non-frontliners). A systematic review by Serrano-Ripoll et al. found that working in a high-risk environment was associated with various mental health problems [39]. Therefore, increasing the number of staff in high-risk environments and reducing their workload would be necessary, as well as establishing a salary gradient between frontliners and non-frontliners.

### Burnout

This study, conducted in 2022, showed that 17.1%, 17.1%, and 22.9% of hospital workers, physicians, and nurses experienced burnout based on the Japanese version of the MBI-GS. Using the MBI, other studies showed the prevalence of burnout – Spanish healthcare workers (15–82%) [48], Italian healthcare workers (25–53%) [49], and Wuhan healthcare workers (13–61%) [50]. We believe that the difference in the prevalence of burnout between these studies was influenced by the differences in the year of investigation, index of burnout, situation of the COVID-19 pandemic, and medical care system during the COVID-19 pandemic. Regarding the study of burnout among hospital workers using the Japanese version of the MBI-GS, a study in April 2020 showed that 31.4%, 13.4%, and 46.8% of hospital workers, physicians, and nurses, experienced burnout, respectively [29]. Moreover, another study, which was conducted after the first wave of the COVID-19 pandemic in Japan, showed that 22.6%, 9.8%, and 29.4% of hospital workers, physicians, and nurses experienced burnout, respectively [31]. The difference in the prevalence of burnout among hospital workers in Japan may be influenced by the differences in the year of investigation and the situation of the COVID-19 pandemic. Additionally, our hospital provided the regular message of protection, comfort, and appreciation from the director and infection control team (ICT) of the hospital, updated information about the virus through the top page of the electronic health record system, quick opening of the consultation service for staff, and hotels for those who could not return home. Furthermore, the hospital mailed a package with a message from the director of the nursing department as well as advice on self-care during the break from the ICT for those who were absent from work due to the COVID-19 infection. During the period when eating out was restricted, the director invited food trucks for lunch breaks, which was well received by the staff. Therefore, these efforts may have contributed to the low burnout rate among hospital workers.

Throughout the COVID-19 pandemic, hospital workers experienced various stresses, such as increased workload and performing new task, which was usually not done [45]. Burnout represents a great concern for healthcare staff working in a large tertiary hospital during the COVID-19 pandemic, and its impact is more burdensome for young workers [29]. Here, emotional exhaustion was also greater in workers in their 20s than in those aged ≥ 40 years. This might be related to the fact that young workers are less familiar with infection control and protective measures and have less experience dealing with extreme events such as a pandemic [51]. Hospital workers with more clinical experience amounts to being better prepared for epidemics, which, in turn, may be conducive to enhanced stronger self-regulation ability [52]. Over time, they might also have come to develop individual coping strategies for increased workloads.

Overall, the findings of this study, combined with the results obtained from the same hospital population during the first pandemic wave [4], suggest that the psychological reaction of hospital care workers to the challenge posed by the COVID-19 pandemic differs according to the specific stage of the pandemic. At the beginning of the first wave, an ‘acute stress’ reaction was observed, which was characterized by a posttraumatic response, fear of contagion, and anxiety [14]. Two years after the pandemic onset, after having dealt with difficult working conditions determined by the sixth pandemic waves, a ‘chronic stress’ reaction appears to have emerged, characterized by increased exhaustion and workload. Therefore, healthcare systems will need to address the pandemic’s psychological impact on hospital care workers by monitoring reactions and performance, paying careful attention to assignments and schedules, assessing occupational risk, and providing psychological support services for those in greater need of mental healthcare [53].

This study had some limitations. The first limitation of our study is the non-response bias as a result of the 22.5%. The response rate was low for both surveys (29.5%, and 22.5%, respectively). However, the results of this study were more consistent with previous studies and seemed to reflect the mental health of hospital workers. Another possibility is that health care workers may be more stressed and have higher scores on the IES-R than the results of the current study because exhausted people are less likely to respond. Second is the external validity as the present study was conducted at a single facility. However, the present results have commonality with the studies conducted in other cities in Japan [29, 31, 54]. They showed that female, nurse compared with doctors felt more event-related distress and burnout.This commonality may indicate that the present results have external validity in some way. Third, we did not assess other common mental states such as depression. However, IES-R total score were associated with depressive symptoms in a previous study [55], and the IES-R scores may reflect the mental health of the staff. Fourth, this was a two-point cross-sectional study targeting different populations while they were employed at the same hospital. Two points in 2020 and 2022 under different COVID-19 situations with different populations cannot be used to accurately extrapolate long-term influence on mental health. Fifth, although we identified four categories (F1 - F4) as significant factors in the previous study, the association between the four categories and job outcomes, such as absence from work, has not been investigated. A systematic review by Meredith et al. found that workplace factors such as workload, work/life balance, job autonomy, and perceived support from leadership were strongly associated with the risk of burnout [56]. In this study, workload and feeling of being protected were also identified as significant factors, work/life balance and job autonomy should be investigated in a future study.

In this pandemic, COVID-19 treatment has been mainly provided by large hospitals such as ours. Therefore, it is important to quickly disseminate a package of solid infection control knowledge, techniques, and equipment to private clinics as well as to share the workload between hospitals and clinics. Furthermore, the burden of COVID-19 treatment, which was placed on a limited number of hospitals, may be problematic; therefore, management is needed among hospitals and clinics to ensure that stress is not placed only on those hospitals involved in COVID-19 treatment.

## Conclusion

Exhaustion and workload among hospital workers have further deteriorated 2 years after the outbreak of the COVID-19 pandemic. Additionally, hospital workers are under unprecedented strain because of the long duration of the pandemic. This study showed that women, those in their 20s, nurses, and frontline workers have a high risk of experiencing stress and burnout. Therefore, health and medical institutions should closely monitor the physical and psychological health of their staff members.

## Data Availability

The dataset supporting the conclusion of the current study is available from the corresponding author upon reasonable request.

## Abbreviations

CFA: confirmatory factor analysis
COVID-19: Coronavirus Disease 2019
DMAT: Disaster Medical Assistance Team
ICT: Infection Control Team
IES-R: Impact of Event Scale-Revised
MBI-GS: Maslach Burnout Inventory-General Survey
PTSD: Posttraumatic Stress Disorder
SARS: Severe Acute Respiratory Syndrome
SD: Standard Deviation
